# MiR-126-3p Is Dynamically Regulated in Endothelial-to-Mesenchymal Transition during Fibrosis

**DOI:** 10.3390/ijms22168629

**Published:** 2021-08-11

**Authors:** Nina P. Jordan, Samuel J. Tingle, Victoria G. Shuttleworth, Katie Cooke, Rachael E. Redgrave, Esha Singh, Emily K. Glover, Hafiza B. Ahmad Tajuddin, John A. Kirby, Helen M. Arthur, Chris Ward, Neil S. Sheerin, Simi Ali

**Affiliations:** 1 Theme-Immunity and Inflammation, Faculty of Medical Sciences, Newcastle University, Newcastle upon Tyne NE2 4HH, UK; ninanina.jordan@gmail.com (N.P.J.); samjamestingle@gmail.com (S.J.T.); victoria.shuttleworth@newcastle.ac.uk (V.G.S.); kcooke@lincoln.ac.uk (K.C.); Emily.glover@newcastle.ac.uk (E.K.G.); H.B.Ahmad-Tajuddin1@newcastle.edu.my (H.B.A.T.); j.a.kirby@ncl.ac.uk (J.A.K.); chris.ward@ncl.ac.uk (C.W.); neil.sheerin@ncl.ac.uk (N.S.S.); 2 Inserm U1082, F-86000 Poitiers, France; 3 Biosciences Institute, Newcastle University, Newcastle upon Tyne NE1 3BZ, UK; rachael.redgrave@ncl.ac.uk (R.E.R.); esha.singh@ncl.ac.uk (E.S.); helen.arthur@ncl.ac.uk (H.M.A.)

**Keywords:** endothelial-to-mesenchymal transition, fibrosis, microRNA, miR-126

## Abstract

In fibrotic diseases, myofibroblasts derive from a range of cell types including endothelial-to-mesenchymal transition (EndMT). Increasing evidence suggests that miRNAs are key regulators in biological processes but their profile is relatively understudied in EndMT. In human umbilical vein endothelial cells (HUVEC), EndMT was induced by treatment with TGFβ2 and IL1β. A significant decrease in endothelial markers such as VE-cadherin, CD31 and an increase in mesenchymal markers such as fibronectin were observed. In parallel, miRNA profiling showed that miR-126-3p was down-regulated in HUVECs undergoing EndMT and over-expression of miR-126-3p prevented EndMT, maintaining CD31 and repressing fibronectin expression. EndMT was investigated using lineage tracing with transgenic Cdh5-Cre-ERT2; Rosa26R-stop-YFP mice in two established models of fibrosis: cardiac ischaemic injury and kidney ureteric occlusion. In both cardiac and kidney fibrosis, lineage tracing showed a significant subpopulation of endothelial-derived cells expressed mesenchymal markers, indicating they had undergone EndMT. In addition, miR-126-3p was restricted to endothelial cells and down-regulated in murine fibrotic kidney and heart tissue. These findings were confirmed in patient kidney biopsies. MiR-126-3p expression is restricted to endothelial cells and is down-regulated during EndMT. Over-expression of miR-126-3p reduces EndMT, therefore, it could be considered for miRNA-based therapeutics in fibrotic organs.

## 1. Introduction

Ischaemia reperfusion injury (IRI) is a well-known phenomenon in many clinical settings, with an interruption in blood flow and tissue oxygenation, such as during acute myocardial infarction, stroke and transplant rejection. IRI at the time of organ transplantation initially manifests as delayed graft function (DGF) [1]. However, several studies have reported that early graft dysfunction carries significant early mortality and those who survive the early post-operative period have a higher propensity to develop fibrosis e.g., bronchiolitis obliterans syndrome (BOS) in lungs [2] or chronic kidney disease [3,4,5]. A comprehensive understanding of molecular events involved in the progression of fibrosis is crucial in formulating prevention strategies.

Fibrosis is associated with disruption of organ architecture and function due to an accumulation of extracellular matrix proteins. These are predominantly produced by activated fibroblasts known as myofibroblasts that are persistent in the context of fibrosis [6]. Myofibroblasts derive not only from endogenous tissue fibroblasts but can also be derived by local differentiation from other cell types [7]. This includes endothelial cells and differentiation towards myofibroblasts is initiated by the process of endothelial-to-mesenchymal transition (EndMT) which can be induced by transforming growth factor family proteins [8,9]. This EndMT results in the loss of endothelial markers and the expression of mesenchymal markers. A contribution of EndMT to the fibroblast pool was observed in cardiac [10], renal [11], pulmonary [12] and intestinal fibrosis [13]. Lineage tracing of endothelial cells using *Tie1*Cre; *R26R*stoplacZ mice showed that 27% to 35% of fibroblasts derived from an endothelial origin in a pre-clinical model of cardiac fibrosis [10]. In the context of kidney fibrosis in the ureteral obstruction mouse model, 10% of fibroblasts appeared to be of endothelial origin using the *Cdh5*-Cre; YFPf/f line for lineage tracing [11]. Thus stabilizing endothelial cells and preventing EndMT could reduce pathological fibrosis.

Accumulating evidence suggests that miRNAs are key regulators of EndMT with the capacity to target several mRNAs involved in the process. MiRNAs are small non-coding RNAs, which regulate biological processes through the repression or degradation of their target mRNA. Up-regulation of miR-21-5p [14] and down-regulation of miR-29 [15] were observed in endothelial cells undergoing EndMT in fibrotic tissues. In endothelial cells, over-expression of miR-21-5p initiated the process of EndMT in the absence of TGFβ implying that miR-21-5p can independently induce EndMT [14]. In contrast, inhibition of miR-21-5p did not affect radiation-induced EndMT in human pulmonary endothelial cells, suggesting that miR-21-5p is not essential for EndMT [16].

Furthermore, some studies have demonstrated that existing treatments for diseases with associated fibrosis influence miRNA expression and so inhibit EndMT [17]. Specifically, atorvastatin acts on the KLF4/miR-483/CTGF axis in Kawasaki’s disease and the downregulation of miR-29 and let-7 observed in diabetic mouse kidneys was prevented by linagliptin and imidrapil, respectively. However, no previous research has focused on cardiac or renal fibrosis.

MiRNA are stable molecules that can be delivered therapeutically offering possible opportunities for regulating biological processes such as EndMT in vivo. For this reason, we developed an in vitro model of EndMT to assess the profile of miRNA using Nanostring technologies. EndMT in vivo was investigated using lineage tracing in two established models of fibrosis: cardiac ischaemic injury and kidney ureteral occlusion injury. Using these models, we have shown that miR-126-3p was significantly down-regulated in cells undergoing EndMT as well as in human renal fibrotic tissues.

## 2. Results

### 2.1. TGFβ2 and IL1β Treatment Induces EndMT in Primary Endothelial Cells

**Figure 1:**
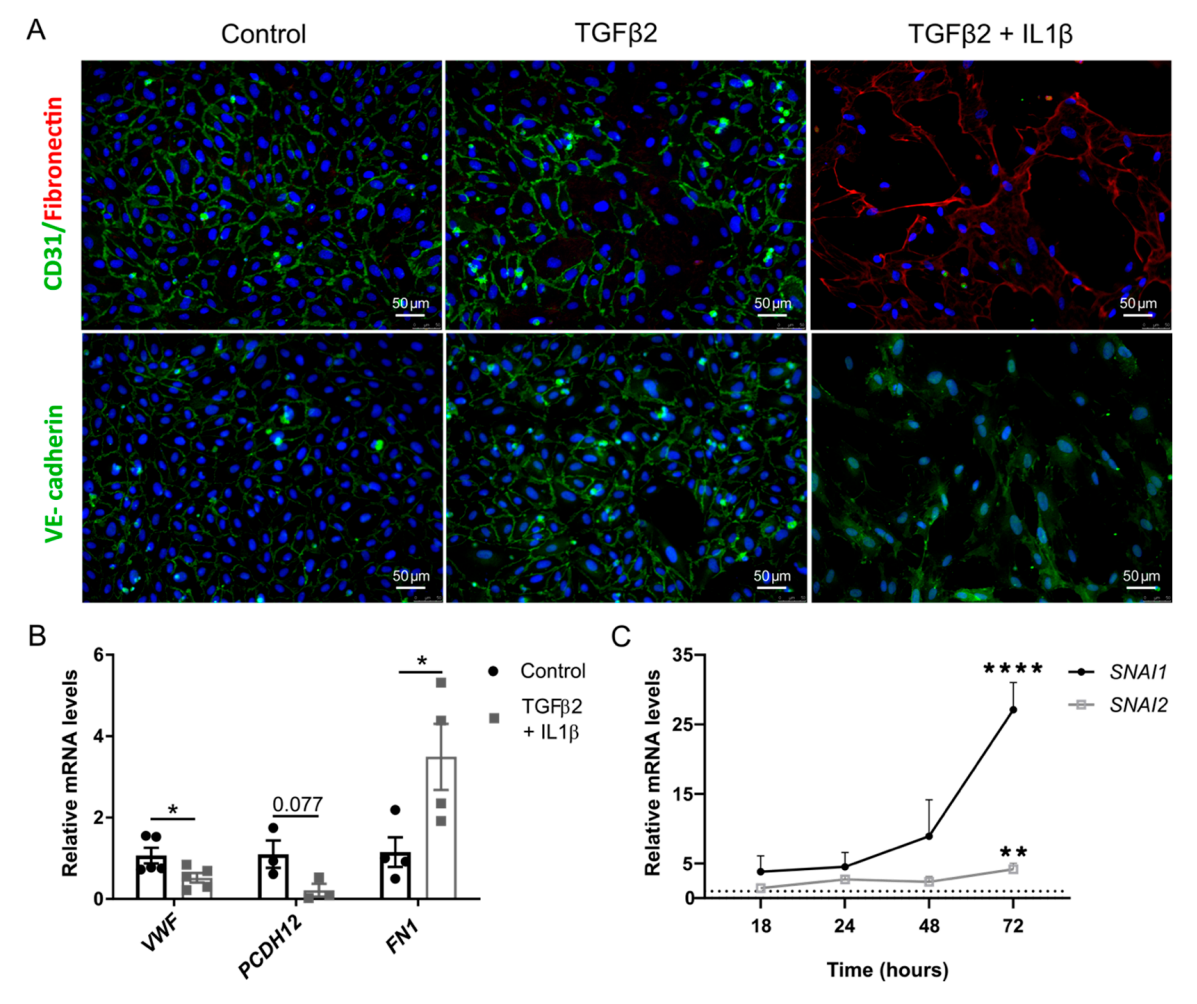
Endothelial cells undergo endothelial to mesenchymal transition with TGFβ2 and IL1β treatment. HUVECs were treated with TGFβ2 (10 ng/mL) or TGFβ2 and IL1β (10 ng/mL) (**A**) Cells were stained post-treatment for the endothelial markers CD31 and VE-cadherin and a mesenchymal marker fibronectin (*n* = 3) (**B**) Gene expression level for vWF (*n* = 5), VE-cadherin 2 (*n* = 3) and fibronectin (*n* = 4) was assessed in endothelial cells 48 h post-treatment with TGFβ2 and IL1β. (**C**) Snail and Slug gene expression was evaluated post-treatment with TGFβ2 and IL1β. Gene expression was normalised to the expression of the housekeeping gene Hprt1 and error bars are standard error of the mean (*n* = 3). Statistical significance was calculated by multiple unpaired Student’s *t*-test and one way ANOVA (* *p* < 0.05; ** *p* < 0.01; **** *p* < 0.0001).

To examine whether EndMT could be induced in human umbilical vein endothelial cells (HUVECs) by IL1β and TGFβ2 treatment, endothelial and mesenchymal markers were assessed. Post-treatment with TGFβ2 and IL1β, the monolayer of HUVECs was disrupted and cells took on a spindle-like fibroblast morphology (Figure S1). Following 6 days of treatment with TGFβ2 or TGFβ2 and IL1β, cells were stained for endothelial junction proteins VE-cadherin, CD31 and the mesenchymal marker fibronectin (Figure 1A). In untreated HUVECs, strong staining was observed for CD31 and VE-cadherin. In contrast, reduction in both endothelial markers was observed in TGFβ2 and IL1β treated cells, whereas, fibronectin staining was increased. In addition, redistribution of VE-cadherin from the membrane to the cytoplasm was observed post-treatment.

Quantitative PCR was used to confirm the loss of endothelial-specific gene expression in treated cells. Von Willebrand Factor (vWF) was significantly down-regulated (*p* = 0.020), whereas, fibronectin was significantly up-regulated 48 h post-treatment with TGFβ2 and IL1β (Figure 1B). There was a trend showing a reduction of VE-cadherin 2 (PCDH12) expression (*p* = 0.07). Additionally, EndMT-associated transcription factors Snail (SNAI1) and Slug (SNAI2) were significantly up-regulated post-treatment (Figure 1C).

Similarly, TGFβ2 and IL1β treatment increased fibronectin expression at the protein level in human microvascular endothelial cells (HMEC) and human saphenous vein endothelial cells (HSVEC) (Figure S2). Gene expression in HMEC of fibronectin and Snail were significantly up-regulated, whereas, vWF was significantly down-regulated post-treatment (Figure S3).

These results indicate that EndMT can be robustly and reliably induced in various cell types including HUVEC, HMEC-1 and HSVEC by TGFβ2 and IL1β treatment.

### 2.2. Changes in miRNA Expression in Primary Endothelial Cells Undergoing EndMT

**Figure 2:**
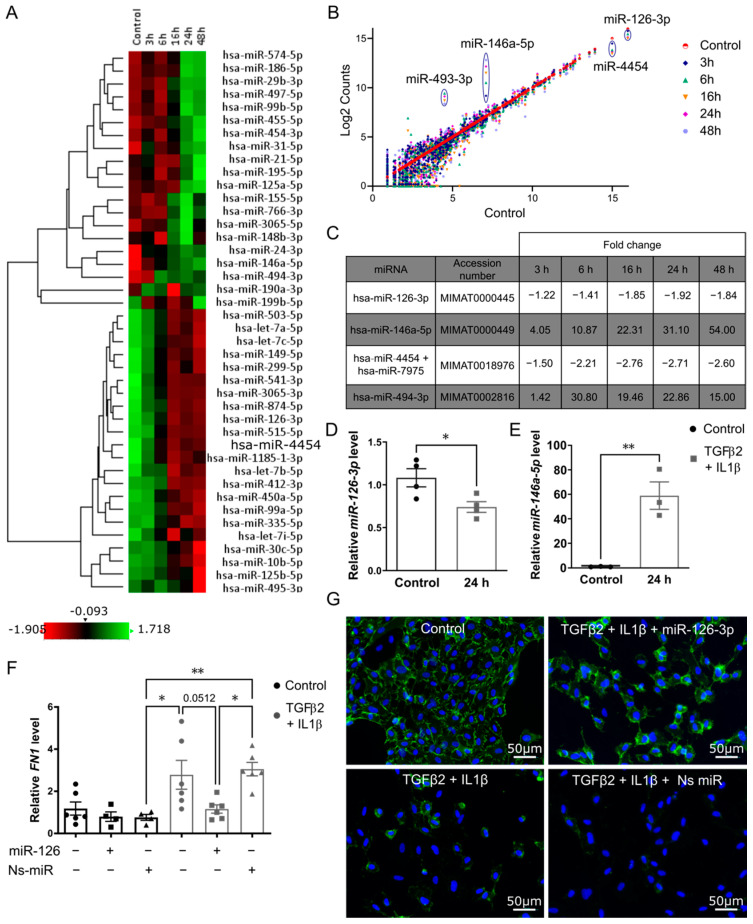
MiR126-3p partially prevents TGFβ2-IL1β-induced EndMT in primary endothelial cells. HUVECs were treated with 10 ng/mL TGFβ2 and IL1β for 3, 6, 16, 24 or 48 h. Total RNA was isolated and miRNAs expression was assessed with the nCounter miRNAs expression panel. (**A**,**B**) Heatmap and scatter plot of the differentially expressed miRNAs in all samples (*n* = 2). Red and green colours represent decreased and increased expression of miRNAs, respectively (**C**) Fold change of miRNAs expression in treated cells compared to control (**D**,**E**) MiR-126-3p (*n* = 4) and miR--146a-5p (*n* = 3) expression by qRT-PCR. Expression of miRNAs was normalized to endogenous controls RNU48 and RNU6, respectively. (**F**) HUVECs were transfected with 50 nM of miR-126-3p mimics using lipofectamine followed by TGFβ2 and IL1β (10 ng/mL) treatment. Transfection of non-specific miRNA (50 nM) was used as the control. Gene expression level of fibronectin was decreased post-transfection in endothelial cells undergoing EndMT. Gene expression was normalised to the expression of the housekeeping gene Hprt1.(*n* = 6) (**G**) Post-transfection, staining for the endothelial marker CD31 showed miR-126-3p promoted retention of an endothelial phenotype in the presence of TGFβ2 and IL1β. Error bars are the standard error of the mean. Statistical significance was calculated by unpaired *t*-test and One way ANOVA (* *p* < 0.05; ** *p* < 0.01).

HUVECs were treated with TGFβ2 and IL1β for 3 h up to 48 h. The profile of miRNAs during EndMT was assessed using an nCounter miRNA assay from Nanostring technologies. From normalised data, a heatmap and a scatter plot were generated to show the differentially expressed miRNA in treated and untreated cells (Figure 2A,B). In Figure 2A hierarchical clustering was performed to group miRNA according to their similarity. For each treatment condition, high and low miRNA counts were illustrated in green and red respectively. Consistent with the literature, miR-21-5p and miR-29b-3p were up-regulated 24 h post-treatment (Figure S4) [14]. MiR-146a-5p was abundant in treated cells and its expression was positively correlated to the time of treatment, increasing over time. Similarly, miR-494-3p was up-regulated post-treatment. Among the more abundant miRNAs in untreated cells, miR-126-3p and miR-4454, were down-regulated post-treatment with TGFβ2 and IL1β (Figure 2C). nCounter assay results were further validated using qRT-PCR which confirmed down-regulation of miR-126-3p and up-regulation miR-146a-5p during EndMT (Figure 2D,E) suggesting their potential role in EndMT.

### 2.3. Over-Expression of miR126-3p Stabilizes Endothelial Phenotype

The role of miR126-3p was previously demonstrated in the context of angiogenesis and vascular integrity. Deletion of this miRNA in mice led to vascular leakage and haemorrhage so miR-126-3p was a logical target for further work [18,19]. To characterize the potential role of miR126-3p in EndMT, a mimic of miR126-3p and non-specific miRNA were transfected for 6 h in HUVECs. After transfection, cells were treated with TGFβ2 and IL1β for 48 h and endothelial cell junction marker CD31 was visualized by immunofluorescence. In cells transfected with non-specific microRNA, TGFβ2 and IL1β treatment led to a reduction in CD31 expression. This reduction was prevented by the overexpression of miR126-3p (Figure 2G). However, miR126-3p overexpression did not prevent the reduction in vWF and VE-cadherin expression in cells treated with TGFβ2 and IL1β (data not shown). Fibronectin gene expression was also assessed in cells transfected with miR126-3p and treated with TGFβ2 and IL1β. MiR126-3p significantly reduced fibronectin gene expression in treated cells in comparison to non-specific microRNA transfected and treated cells (*p* = 0.016) (Figure 2F). Similarly, miR-126-3p over-expression significantly reduced fibronectin gene expression in treated HMEC (Figure S5). Thus, MiR126-3p over-expression reduces but does not completely prevent EndMT.

### 2.4. EndMT Occurs in Cardiac and Renal Fibrosis

**Figure 3:**
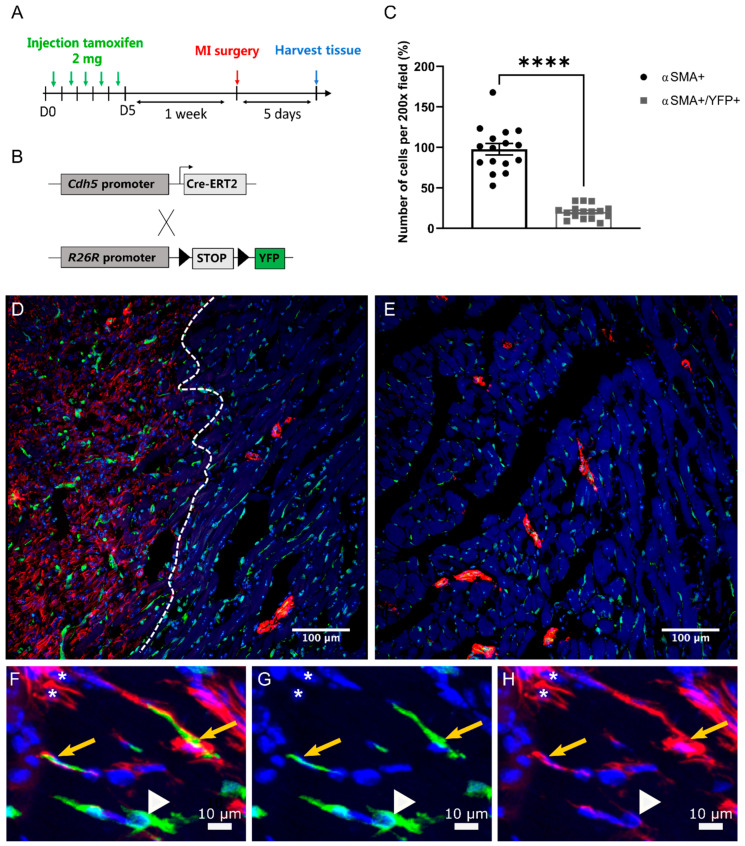
Endothelial-to-mesenchymal transition is present in fibrotic hearts. (**A**) Timeline for transgenic mouse heart surgery and the experiment. (**B**) Schematic representation of Cdh5-CreERT2; Rosa26R-stop-YFP mouse model. The Cdh5-CreERT2 mouse expresses the tamoxifen-activated Cre recombinase under the VE-cadherin promoter, Cdh5. Rosa26R-stop-YFP mice were crossed with inducible Cdh5-CreERT2 to permit Cre-dependent lineage tracing in endothelial cells. Transverse sections of mouse heart post-MI were stained for αSMA and YFP (endothelial cell origin). (**C**) Quantification of YFP+ αSMA+ and αSMA+ only cells from infarcted area of hearts 5 days post-MI. (**D**–**H**) Cdh5-Cre-ERT2; Rosa26R-stop-YFP mice underwent MI and were sacrificed 5 days later. Micrographs show an infarcted area (**D**) and a non-infarcted area (**E**). (**D**) The dotted line indicates the border between the injured (left side) and the non-injured area (right side). A higher magnification of the infarcted area is shown in the lower panel (**F**,**G**). YFP+ αSMA+ cells are indicated with a yellow arrow. Single YFP+ or αSMA+ cells were designated by a white arrowhead or white star respectively (*n* = 4). Statistical significance was calculated by paired *t*-test (**** *p* < 0.0001).

**Figure 4:**
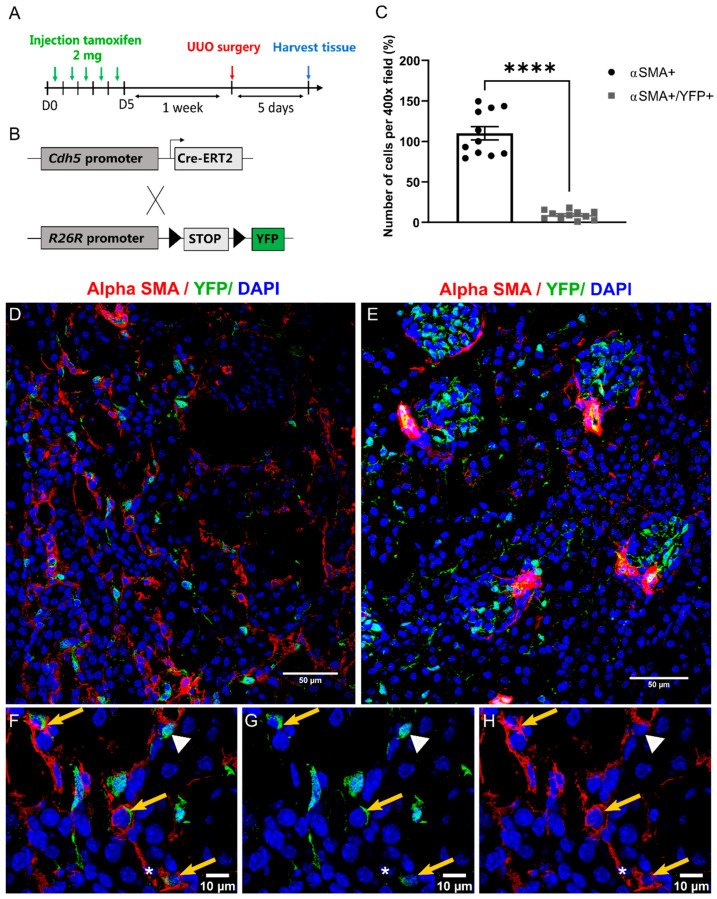
Endothelial-to-mesenchymal transition is present in fibrotic kidneys (**A**) Timeline for transgenic mouse kidney surgery and the experiment. (**B**) Schematic representation of Cdh5-Cre-ERT2; Rosa26R-stop-YFP mouse model. Cdh5-CreERT2 mouse allows the expression of the tamoxifen-inducible Cre recombinase under VE-cadherin promoter Cdh5. Cdh5-Cre-ERT2; Rosa26R-stop-YFP mice underwent UUO and were sacrificed 5 days later. Staining for αSMA and YFP (endothelial cell origin) was performed on healthy or post-UUO mouse kidneys. (**C**) Quantification of YFP+ αSMA+ and αSMA+ only cells in the fibrotic kidneys. (**D**–**H**) Representative image of healthy (**E**), fibrotic kidneys (**D**) and zoom of the fibrotic kidney (**F**,**G**,**H**). Co-stained YFP+ αSMA+ cells were indicated with a yellow arrow. Single YFP+ or αSMA+ cells were designated by a white arrowhead or white star respectively (*n* = 3). Statistical significance was calculated by paired *t*-test (**** *p* < 0.0001).

To determine whether EndMT contributed to cardiac and renal fibrosis, we evaluated the presence of mesenchymal cells of endothelial origin using lineage tracing. The transgenic mouse Cdh5-CreERT2; Rosa26R-stop-YFP expresses a tamoxifen-inducible Cre recombinase specifically in endothelial cells. Activation of Cre-ERT2 with tamoxifen leads to a permanent genetic change specifically in endothelial cells causing them to express the fluorescent marker YFP. This marker can then be used to trace cells with an endothelial origin (Figure 3B and Figure 4B). Following tamoxifen treatment, cardiac surgery was performed to generate myocardial infarction and heart tissue was analysed 5 days post-surgery when myofibroblasts are abundant in the infarcted region (Figure 3A and Figure S6). In Cre negative mice, no YFP cells were observed in Sham-operated or MI hearts, confirming the lack of YFP expression in the absence of Cre activity. However, in Cre positive mice, YFP was found colocalised with CD31 staining in sham hearts confirming the endothelial specificity of this Cre line (Figure S7). Double immunofluorescent staining was performed to detect expression of YFP of endothelial origin together with the mesenchymal marker alpha-smooth muscle actin (αSMA) which would be consistent with the evidence of EndMT (Figure 3D–H). Importantly, 20% of αSMA+ mesenchymal cells were positive for YFP staining indicating they were of endothelial origin and therefore derived by EndMT (Figure 3C). Using another fibroblast marker FAP, double-positive cells for FAP+ YFP+ were also observed in the infarcted area (Figure S8) In contrast, in the remote regions of the heart (distant from the injured area), there were no examples of YFP and αSMA or YFP and FAP colocalization (Figure 3E), suggesting that injury itself generated local signals to drive EndMT.

To detect EndMT in the context of renal fibrosis, Cdh5-CreERT2; Rosa26R-stop-YFP mice underwent unilateral ureteral obstruction and were sacrificed 5 days later (Figure 4A,B). Both kidneys were collected and the right kidney was used as a control for the injured left kidney. In the healthy kidneys (Figure 4E and Figure S9), YFP+ cells were observed in glomeruli, in capillaries and the intima layer of larger blood vessels. In addition, αSMA staining was found in vascular smooth muscle cells (VMSC). In the fibrotic kidney, αSMA was expressed by the VSMC and the myofibroblast population (Figure 4D–H). In fibrotic kidneys, on average 9% of the mesenchymal αSMA+ cells population were dual positive (αSMA+, YFP+, Figure 4C) whereas, no colocalisation of αSMA and YFP staining was found in the healthy kidney (Figure 4E). This suggests that EndMT contributes to the pool of myofibroblasts in renal and cardiac fibrosis.

### 2.5. Expression of miR-126-3p in Normal Cardiac Tissue

**Figure 5:**
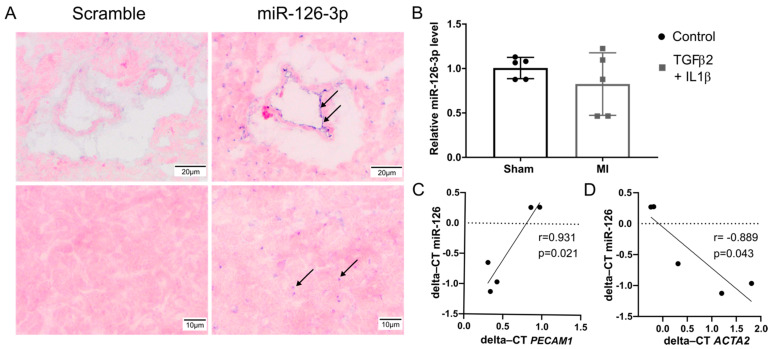
MiR126-3p is expressed in cardiac endothelial cells and is correlated with EndMT. (**A**) In situ hybridisation of miR-126-3p (40 nM) or scramble double-DIG labelled LNA probes (40 nM) was performed on frozen sections of the healthy mouse heart. Pictures were focused on vessel and myocardium (*n* = 3). (**B**) Analysis of miR126-3p in sham and MI heart was assessed by RT-qPCR. Expression was normalised to the expression of the housekeeping gene SnoRNA 202 and errors bars are standard error of the mean. (*n* = 5). (**C**) Correlation of CD31 gene expression (PECAM1) to miR-126-3p expression in fibrotic heart. (**D**) Correlation of αSMA gene expression (ACTA2) to miR-126-3p expression in the fibrotic heart. Statistical significance was calculated by unpaired *t*-test.

To examine the expression of miR-126-3p in cardiac tissue, in-situ hybridization was performed on healthy mouse heart sections. Hybridisation with a scrambled sequence probe was performed as the control. MiR126-3p was observed in endothelial cells, in the intima of larger blood vessels and intramuscular capillaries, whereas, hybridisation with the control probe did not show any staining (Figure 5A).

To investigate the expression of miR-126-3p in cardiac fibrosis, we analysed samples of the murine left ventricle 7 days after surgically induced myocardial infarction versus sham operated mice. Expression of miR-126-3p was similar in MI and uninjured hearts (Figure 5B). However, varying levels of fibrosis were observed as the whole left ventricle was used to analyse miR-126-3p expression. To normalize for the variation in fibrosis, the expression of miR-126-3p was compared to the expression of CD31 and the mesenchymal marker αSMA. A statistically significant positive correlation was seen between miR-126-3p and CD31 gene expression (Figure 5C), and a significant negative correlation between miR-126-3p and the gene expression of the mesenchymal marker αSMA (Figure 5D). Thus, hearts with higher levels of miR-126-3p had lower αSMA expression.

#### Expression of miR-126-3p in Normal Renal Tissue

**Figure 6:**
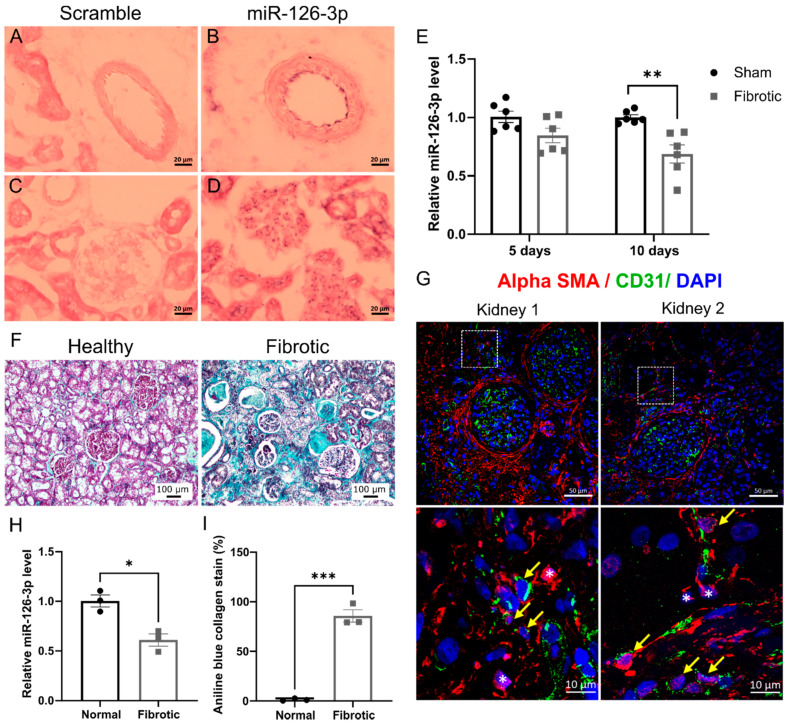
MiR-126-3p is expressed in renal endothelial cells and is down-regulated in fibrotic kidneys. (**A**–**D**) In situ-hybridisation was performed on frozen sections of healthy mouse kidneys with 40 nM of miR-126-3p or scramble double-DIG labelled LNA probes. The micrographs show blood vessel (**A**,**B**) or glomeruli (*n* = 2) (**C**,**D**). (**E**) RT-qPCR analysis of miR-126-3p in sham operated, 5 and 10 days post-UUO kidneys. Expression was normalised to the expression of the housekeeping gene SnoRNA 202 (*n* = 6). (**F**) Masson trichrome staining in human normal and fibrotic kidneys (light blue staining indicates connective tissue staining). (G) Sections of human fibrotic kidneys were stained for αSMA and CD31. A higher magnification (white square) is shown in the lower panel for each kidney. Co-stained CD31+αSMA+ cells were indicated with a yellow arrow. Single αSMA+ cells were designated by white stars. (**H**) Expression of miR126-3p was assessed by RT-qPCR in normal and fibrotic human kidneys. Expression was normalised to the expression of the housekeeping gene RNU48 and errors bars are standard error of the mean (*n* = 3). (**I**) Quantification of aniline blue collagen stain in Masson trichrome human kidneys sections (*n* = 3). Statistical significance was calculated by unpaired *t*-test. (* *p* < 0.05; ** *p* < 0.01; *** *p* < 0.001).

To investigate the localisation of miR-126-3p in normal kidneys, in-situ hybridisation was performed on frozen healthy kidney sections using the miR-126-3p probe as well as a scrambled probe to ensure the lack of non-specific staining. In-situ hybridisation revealed the presence of miR-126-3p in the intima layer of the larger blood vessel (Figure 6B) and the glomerular capillaries (Figure 6D). No staining was observed when a scrambled probe was used (Figure 6A–C).

To further determine the level of expression of miR-126-3p in renal fibrosis, we used a murine UUO model. MiR-126-3p expression was down-regulated in UUO kidneys compared to contralateral control kidneys, which reached statistical significance at day 10 (Figure 6E). To confirm whether downregulation of miR-126-3p expression was also seen in human fibrotic tissue, the unaffected pole of human kidneys removed during resection of small renal tumours were screened to characterise the level of fibrosis and assigned to cohorts of fibrotic and non-fibrotic tissue. The level of fibrosis was assessed by the quantification of aniline blue collagen in Masson trichrome staining (Figure 6F,I). Double immunofluorescent staining was performed for CD31 and αSMA to assess EndMT in human fibrotic kidneys (Figure 6G).

In both fibrotic kidneys, a population of cells were CD31+αSMA+ indicating that myofibroblasts could derive from endothelial cells in human kidney fibrosis. Using qPCR miR-126-3p was significantly down-regulated in fibrotic human kidneys compared to normal kidneys (Figure 6H). This suggests that miR-126-3p expression is decreased as a result of EndMT occurring during kidney fibrosis.

## 3. Discussion

Fibrosis is characterised as a pathologic tissue repair due to an accumulation of extracellular matrix primarily secreted by myofibroblasts. Different sources of myofibroblasts have been reported including the process of endothelial-to-mesenchymal transition. The full understanding of the contribution of EndMT to fibrosis has suffered from a lack of precise functional and molecular definition and robust human data corroborating the extent of EndMT in human subjects. MiRNAs play a key role in regulating EndMT by targeting multiple components associated with signalling pathways that regulate EndMT. Although our current understanding of the molecular mechanisms underlying the miRNA-EndMT axis is advancing, more work is required to better understand this complex network.

In this study, we provide evidence from in vitro, preclinical animal models of disease in two organs and from complimentary assessment of human kidney tissue that miR-126-3p is expressed in endothelial cells and a reduction in expression is associated with loss of endothelial phenotype. These findings suggest that miR-126-3p is a marker of endothelial integrity in fibrotic disease. In addition, manipulation of miR-126-3p can stabilize endothelial phenotype and identifies this microRNA as a possible therapeutic target for endothelial regeneration in fibrosis.

To study EndMT in vitro, primary human umbilical vein endothelial cells were treated with TGFβ2 and IL1β. Endothelial markers such as VE-cadherin, CD31 and vWF decreased and mesenchymal markers such as fibronectin increased. Furthermore, Snail and Slug were up-regulated post-treatment. Numerous models of EndMT have been generated in HUVECs with the induction of oxidative stress, treatment with TGFβ2 alone or combined with IL1β [8,14]. Maleszewska et al. demonstrated that the combination of TGFβ2 and IL1β led to synergistic induction of EndMT in HUVEC. In addition, the transcription factors Snail and Slug are known to be involved in the process of EndMT and silencing of Snail prevented EndMT in endothelial cells treated with TGFβ1 [20]. Our results are consistent with the up-regulation of Snail and Slug previously observed in endothelial cells undergoing TGFβ1-induced EndMT or hypoxia-induced EndMT [21,22].

To investigate the miRNA profile associated with EndMT, we performed an nCounter assay in endothelial cells undergoing EndMT. This showed the differential expression of various miRNAs including miR-146a-3p, miR-126-3p and miR-494-3p. Interestingly, mi-R126-3p was highly expressed in HUVEC [23] and participates in the regulation of angiogenesis and vascular integrity. In vitro overexpression of miR-126-3p in the human saphenous vein endothelial cells results in increased migration and proliferation by the silencing of the validated targets SPRED1 and PiK3R2 [24]. Additionally, knockdown of miR-126-3p in zebrafish led to vascular leakage and haemorrhage, pointing to its importance in maintaining endothelial integrity [18,19]. The down-regulation of miR-126-3p in endothelial cells undergoing transition associated with the loss of their endothelial characteristics suggests that miR126-3p also has a role in the regulation of endothelial-to-mesenchymal transition.

In our study, miR-126-3p transfection led to the maintenance of CD31 expression and the reduction of fibronectin expression in HUVEC undergoing EndMT. However, over-expression of miR-126-3p did not affect vWF expression. These results demonstrate a partial role of miR-126-3p in inhibiting EndMT. In rat endothelial progenitor cells treated with TGFβ1, over-expression of miR-126-3p led to the down-regulation of the mesenchymal marker αSMA and tended to restore the endothelial progenitor marker CD34 [25].

In the study by Kumarswamy et al., partial prevention of EndMT was observed with the transfection of a miR-21-5p antagomir. MiR-21-5p was up-regulated in TGFβ2-induced EndMT, and its inhibition results in the restoration of VE cadherin and the reduction of FSP1 protein expression. Interestingly, miR-21-5p was significantly up-regulated in our EndMT model. These results suggest that several miRNAs may act synergistically to modulate EndMT and could contribute to new therapeutic strategies in miRNA-based therapy.

Lineage tracing of endothelial cells has been used as a tool to elucidate the role of EndMT in fibrosis. However, many controversies exist about the contribution of EndMT to the myofibroblast pool. In cardiac fibrosis, EndMT was observed in hearts post-aortic banding with lineage tracing of endothelial cells using the promoter of Tie1 while lineage tracing using the promoter of VE-cadherin (*Cdh5*) did not show any contribution of EndMT to cardiac fibrosis [26]. In the context of kidney fibrosis, approximately 10% of αSMA positive cells were of endothelial origin [11]. Interestingly, Curci et al**.** have demonstrated that the process of EndMT and vascular rarefication at renal levels are activated by IRI through priming of the complement system and subsequent activation of the AKT pathway leading to renal fibrosis [27]. In further support of the occurrence of EndMT in fibrosis, a strong association was observed between EndMT markers and poor recovery of renal allografts in patients with acute tubular necrosis [28]. To assess the contribution of EndMT in fibrosis, we used lineage tracing of endothelial cells. This was performed using transgenic mice with the tamoxifen-inducible CreERT2 system. This system allows the performance of the lineage tracing in a specific time and space, thus detecting only de-novo EndMT in fibrotic tissues.

Our use of *Cdh5*-CreERT2; *Rosa26R*-stop-YFP mice allowed the inducible expression of YFP specifically in cells of endothelial origin. The presence of EndMT was therefore assessed by double immunofluorescence of αSMA a marker of activated fibroblasts and co-expression of YFP, denoting endothelial cells. In both the fibrotic kidneys and fibrotic heart, double-positive cells (αSMA positive and YFP positive) were seen, implying the presence of EndMT. No co-localization was observed in sham-operated heart and control kidneys. As VSMC are αSMA+, large vessels were excluded for the quantification of EndMT to focus on the transition of endothelial cells to myofibroblasts, however, this does not exclude VSMC from muscularized arterioles. A combination of commonly used markers of fibroblast would be required to further characterize the population of mesenchymal cells subjected to EndMT. In concordance with the literature, our results confirm a contribution of EndMT to the mesenchymal population both in cardiac and kidney fibrosis. In our study, 20% and 9% of αSMA positive cells were of endothelial origin in cardiac and kidney fibrosis respectively, which is consistent with previous studies.

MiR-126-3p was down-regulated in vitro in HUVEC undergoing EndMT and EndMT was also observed in cardiac and renal fibrosis. Based on these results, we investigated the expression of miR126-3p in vivo. In situ hybridisation of miR-126-3p was performed in healthy heart and kidney from mice. We showed for the first time that expression of miR126-3p was confined to endothelial cells in healthy heart, the glomeruli and the intima of the blood vessels in the kidney. This suggests that miR-126-3p is restricted to endothelial cells in both cardiac and renal tissue.

We further assessed the expression of miR-126-3p in mouse fibrotic tissue. In heart post-MI, miR-126-3p tends to decrease compared to sham-operated mice. In sections of left ventricle post-MI, miR-126-3p positively correlated with CD31 and negatively correlated with αSMA, implying that miR-126-3p is decreased in cardiac fibrosis. In renal fibrotic tissue, relative quantification revealed a significant down-regulation in mice post-UUO. Consistent with these results, a significant down-regulation of miR-126-3p was observed in human fibrotic kidney tissue compared with non-fibrotic tissue. Capillary rarefaction is one of the characteristic features of progressive fibrotic disease. In targeting EndMT, it may be possible to reduce both the accumulation of fibrotic tissue and the loss of capillaries to improve organ function [29,30]. Recent data suggests both cell free-miR-126 and EV-miR-126 as a negative regulation biomarker for acute myocardial infarction and cardiovascular disease. In addition, a study from Lovisa et al. showed that EndMT was responsible for vascular leakage in kidney fibrosis and as previously discussed, neonatal miR126^−/−^ mice are susceptible to vascular leakage and develop with multiple vascular integrity defects [18,31]. Therefore, miR-126-3p could be involved in the EndMT-induced vascular leakage.

In the context of ischemia-reperfusion injury, a diminution of miR-126-3p was observed in correlation with endothelial cell damage and reduced kidney function in human kidneys undergoing a period of reperfusion [32]. Furthermore, overexpression of miR-126 in the hematopoietic compartment augments neovascularization in subcutaneously implanted Matrigel plugs and protects the kidney from IRI. This data supports a direct causal role for miR-126 augmented vasculogenesis leading to preservation of renal function following IRI [33].

In conclusion, miR-126-3p levels are diminished in fibrotic tissue; a response that is conserved across multiple organs and species. We showed that increasing the level of miR-126-3p in endothelial cells partially prevented EndMT, further supporting the evaluation of a potential therapeutic role. Our findings suggest that the contribution of EndMT to the fibroblast population in fibrotic disease might be reduced by overexpression of miR-126-3p, therefore slowing the progression of fibrosis. In addition, miR-126-3p was exclusively localised to endothelial cells in the heart and kidney, which makes this miRNA an attractive target for future therapy. Additionally, the stable nature of miRNA makes them an attractive candidate for biomarkers. Understanding changes in miR-126-3p levels in fibrosis could therefore contribute to future clinical care through diagnosis and monitoring of disease.

## 4. Materials and Methods

### 4.1. Cell Culture

HUVECs were obtained from pooled donors (Promocell). Cells were maintained in endothelial cell growth medium (Promocell) supplemented with 1% penicillin/streptomycin. Cells were used between passages 2 and 7. Human recombinant TGFβ2 and human recombinant IL1β were purchased from Prepotech and were used at a final concentration of 10 ng/mL.

HUVECs were seeded in chamber slides 24 h prior to the transfection of miRNA mimics. Transfections were carried out in OPTI-MEM media for 6 h with 50 nM miRIDIAN microRNA hsa-miR-126-3p mimics (Dharmacon) using lipofectamine RNAimax (ThermoFisher) as transfection reagent according to manufacturer’s protocol. Non-specific mirVana miRNA mimic (50 nM) (ThermoFisher) was used as a negative control for the transfection. Post-transfection, cells were treated with TGFβ2 and IL1β for 48 h.

### 4.2. Animals

All in vivo procedures were conducted in accordance with the Guidance on the Operation of the Animals (Scientific Procedures) Act, 1986 (UK Home Office) and with approval from the local ethics committee Cdh5-Cre-ERT2; Rosa26R-YFP floxed stop mice were generated by intercrossing Cdh5-Cre-ERT2 [34] and Rosa26R-YFP floxed stop Cre reporter mice [35]. Cre negative mice were used as the control. Prior to surgery, each mouse was given one injection per day for 5 consecutive days of 2 mg of tamoxifen (Merck) resuspended in peanut oil (Merck).

### 4.3. Animal Procedures

Acute myocardial infarction (MI) was generated in adult C57BL/6 mice (14 to 17 weeks of age) as previously described [36]. Fentanyl/fluanisone (‘Hypnorm’, 0.4 mL/kg) was administrated to mice for analgesia and mice were anaesthetised with isoflurane (3% isoflurane/97% oxygen). A left thoracotomy was performed to expose the heart and the left anterior descending artery which, was permanently ligated using a 7-0 prolene suture. Post suture blanching of the myocardium distal to the ligature was used to confirm MI. Mice in the sham group underwent left-side thoracotomy without artery ligation. Buprenorphine (Vetergesic, 0.05 mg/kg) was injected subcutaneously for postoperative analgesia and mice were culled 7 days post-surgery.

Kidney fibrosis was induced by Unilateral Ureteral Obstruction (UUO). C57BL/6 mice aged 8–10 weeks were anaesthetised with isoflurane and subjected to UUO. Laparotomy was performed to expose the kidney and the left ureter was ligated on the left kidney. The non-ligated kidney was used as a control. Mouse kidneys were harvested at 5 and 10 days post-UUO with 6 animals per group.

### 4.4. Human Kidney Tissue

The Newcastle Institute of Transplant Tissue Biobank (Newcastle and North Tyneside Research Ethics Committee 1,17/NE/0022) stores renal tissue samples from the non-cancerous pole of human kidneys resected for small renal tumours, to generate a bank of renal tissue. Approval was obtained from The Newcastle Institute of Transplant Tissue Biobank to research these samples. The unaffected poles of tumour nephrectomies were examined for the presence of interstitial fibrosis and tubular atrophy (IFTA). Kidneys with no IFTA were classed as non-fibrotic and those with IFTA as fibrotic. Quantification of miR-126-3p was performed with PCR as described below. Standard Masson Trichrome stains were performed to illustrate differences in the level of fibrosis.

### 4.5. Immunofluorescent Cytostaining

Cells were grown in a chamber in a humidified atmosphere of 5% CO_2_ at 37 °C and fixed with cold methanol (−20 °C) at room temperature. Slides were treated with blocking solution (PBS 1%BSA) and were incubated with primary antibodies overnight at 4 °C prior to applying fluorescent conjugated secondary antibodies for one hour. Primary antibodies to VE-cadherin (1:200, ab33168, Abcam, Cambridge, UK), CD31 (1:100, 550389, BD Pharmingen, San Diego, CA, USA), Fibronectin (1:200, ab2413, Abcam) were used. Incubation of secondary antibodies in 1:200 dilutions were performed with goat anti-mouse Alexa Fluor 488, goat anti-rabbit Alexa Fluor 488 and goat anti-rabbit DyLight 594. Counterstaining was performed with DAPI (Biolegend, San Diego, CA, USA) prior to mounting with Fluoromount (Merck, Franckfurt, Germany) Slides were analysed using a Zeiss Axio Imager.

### 4.6. Immunofluorescent Tissue Staining

Immunofluorescent staining was performed on frozen tissue sections. Tissues were fixed in 1% paraformaldehyde overnight at 4 °C, equilibrated with 30% of sucrose and frozen in OCT. Sections were fixed in acetone and stored at −20 °C prior to staining. Primary antibodies were applied overnight at 4 °C in a humidified chamber. For lineage tracing, anti-GFP (1:400, ab13970, Abcam) antibody was used to detect YFP, anti-CD31 (1:200, 550274, BD Pharmingen) and anti-CD31 (1:200, ab28364, Abcam) were used to detect endothelial cells, and anti-αSMA conjugated with Cy3 (1:200, C6198, Merck) was used to detect myofibroblasts. Slides were incubated with fluorescently conjugated secondary antibodies for 2 h at room temperature and mounted with DAPI Prolong Gold (ThermoFisher, Scientific, Paisley, UK). Incubation of secondary antibodies in 1:200 dilutions were performed with goat anti-chicken Alexa Fluor 488, goat anti-rat Alexa Fluor 594 and goat anti-mouse Alexa Fluor 488. Tissues were imaged with Nikon A1r+ and Leica SP8 STED confocal microscopes

### 4.7. Real-Time Polymerase Chain Reaction

Total RNA including small RNAs was isolated from tissue using a miRNeasy extraction kit (Qiagen, Hilden, Germany) and from cells using a mirVana Paris RNA extraction kit (ThermoFisher) according to the manufacturer’s instructions. cDNA was synthesised from miRNAs and messenger RNA with TaqMan microRNA reverse transcription kit (ThermoFisher) and Tetro cDNA reverse transcriptase kit (Bioline, London, UK) respectively. Real-time polymerase chain reactions were carried out with TaqMan assay probes or with TaqMan miRNA assay (ThermoFisher).

### 4.8. MiRNA Profile

The miRNA profile was performed using the human v3 miRNA assay from Nanostring technologies. Up to 800 miRNAs were screened according to the manufacturer’s instructions. The expression of each miRNA was normalised to the positive and negative controls, and to the first 100 highest expressed miRNAs. Normalised data were then analysed with nSolver software.

### 4.9. In-Situ Hybridisation

In-situ hybridisations were performed on frozen sections with double-DIG-labelled Hsa-miR-126-3p probe (GCATTATTACTCACGGTACGA) and scramble-miR probe (GTGTAACACGTCTATACGCCCA) (Qiagen) as the negative control. Tissue sections were hybridised at 54 °C with microRNA ISH buffer and 40 nM of the miRNA detection probe. Slides were blocked and incubated with Digoxigenin antibody [21H8] conjugated to alkaline phosphatase (AP) (Roche, Basel, Switzerland) for 1 h. NBT-BCIP substrate was applied for a minimum of an hour at 30 °C. The reaction was stopped with AP stop reaction and sections were counterstained with Nuclear Fast Red (Vector Laboratories Burlingame, CA, USA). Slides were mounted with permanent aqueous mounting medium (DAKO, Glostrup, Denmark) and analysed with an Olympus microscope equipped with an SC50, 5-megapixel colour camera.

### 4.10. Statistical Analysis

Results are presented as mean ± standard deviation unless stated otherwise. Statistical analyses were performed by a two-tailed unpaired *t*-test for the comparison of two groups. For more than 2 groups, one-way ANOVA was carried out followed by Tukey comparison test. Data were analysed with GraphPad prism software and a *p*-value < 0.05 was considered as statistically significant.

## Data Availability

The data presented in this study are available in article entitled “MiR-126-3p Is Dynamically Regulated in Endothelial-to-Mesenchymal Transition during Fibrosis” and associated supplementary files.

## Supplementary Materials

The following are available online at https://www.mdpi.com/article/10.3390/ijms22168629/s1.
