# Supplementary material for: MiR-126-3p Is Dynamically Regulated in Endothelial-to-Mesenchymal Transition during Fibrosis
Source: Int J Mol Sci. 2021 Aug 11;22(16):8629. doi: 10.3390/ijms22168629 (PMC8395326; doi:10.3390/ijms22168629)
Supplement: Supplementary file 1 [file ijms-22-08629-s001.zip › ijms-1310888-supplementary.pdf]

1. *Supplemental Material and Methods*

Cell Culture

The human microvascular endothelial cell line (HMEC-1) was purchased from ATCC and was grown in reconstituted MCDB 131. MCDB 131 was supplemented with 10% Foetal bovine serum (FBS), 100 U/mL penicillin, 100 μg/mL streptomycin, 10 ng/mL of epidermal growth factor (EGF) and 1 μg/mL of hydrocortisone.

Human saphenous vein endothelial cells (HSVEC) were donated within the institute and maintained in cell vascular basal medium supplemented with 50 ug/mL ascorbic acid, 5 ng/mL EGF, 5% FBS, 0.75 U/mL heparin sulphate, 10 mM L- glutamine, 1 ug/mL hydrocortisone and 0.2% bovine brain extract.

2. *Supplemental Figures*

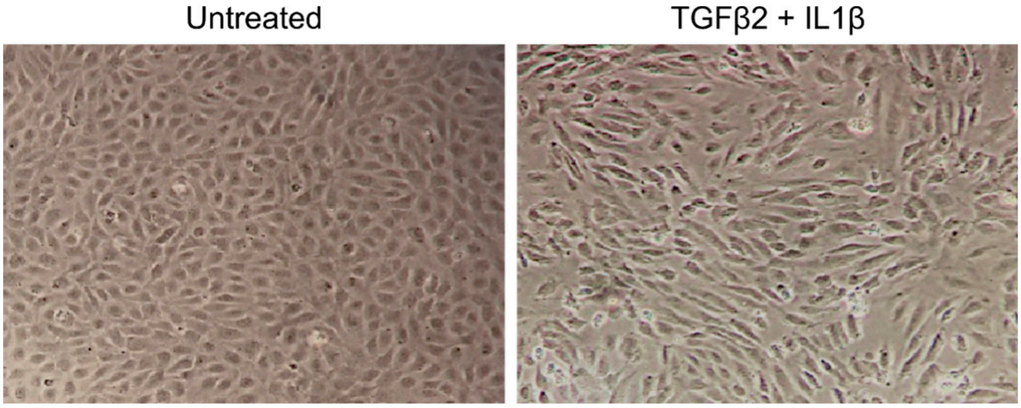

Figure S1. Effect of TGFβ2 and IL1β treatment on HUVEC morphology.

Cells were treated with TGFβ2 (10 ng/mL) and IL1β (10 ng/mL) for 24 h. HUVECs normally grow as a monolayer with a cobblestone appearance. Post-treatment, there is a disruption of the monolayer and cells take on a spindle shape morphology.

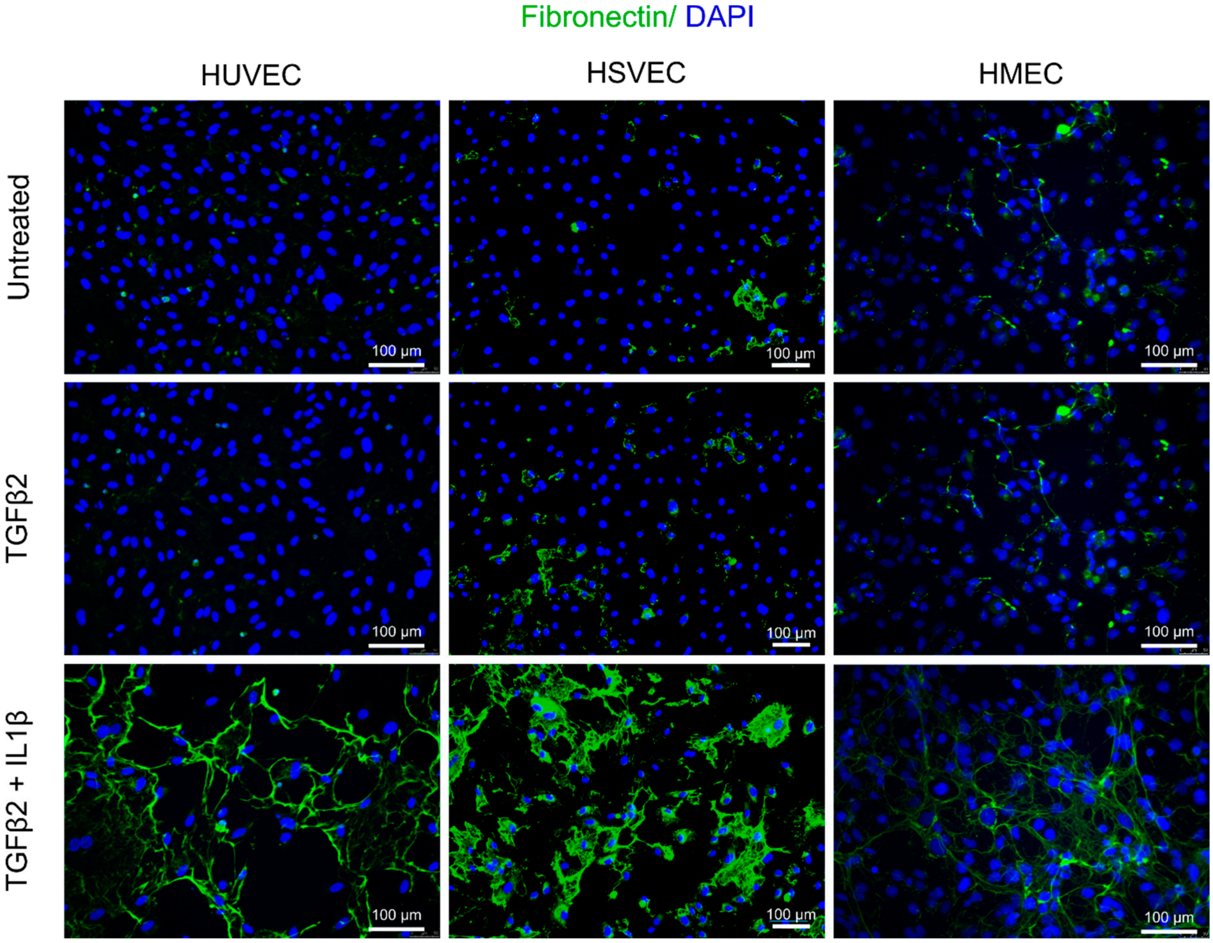

Figure S2. Fibronectin staining in endothelial cells treated with TGFβ2 or TGFβ2 and IL1β.

Human umbilical vein endothelial cells (HUVEC) (n = 4), Human saphenous vein endothelial cells (HSVEC) (n = 3) and Human microvascular endothelial cells (HMEC) (n = 2) were treated for 6 days

with TGFβ2 (10 ng/mL) or TGFβ2 and IL1β (10 ng/mL). Cells were stained for fibronectin and counterstained with DAPI.

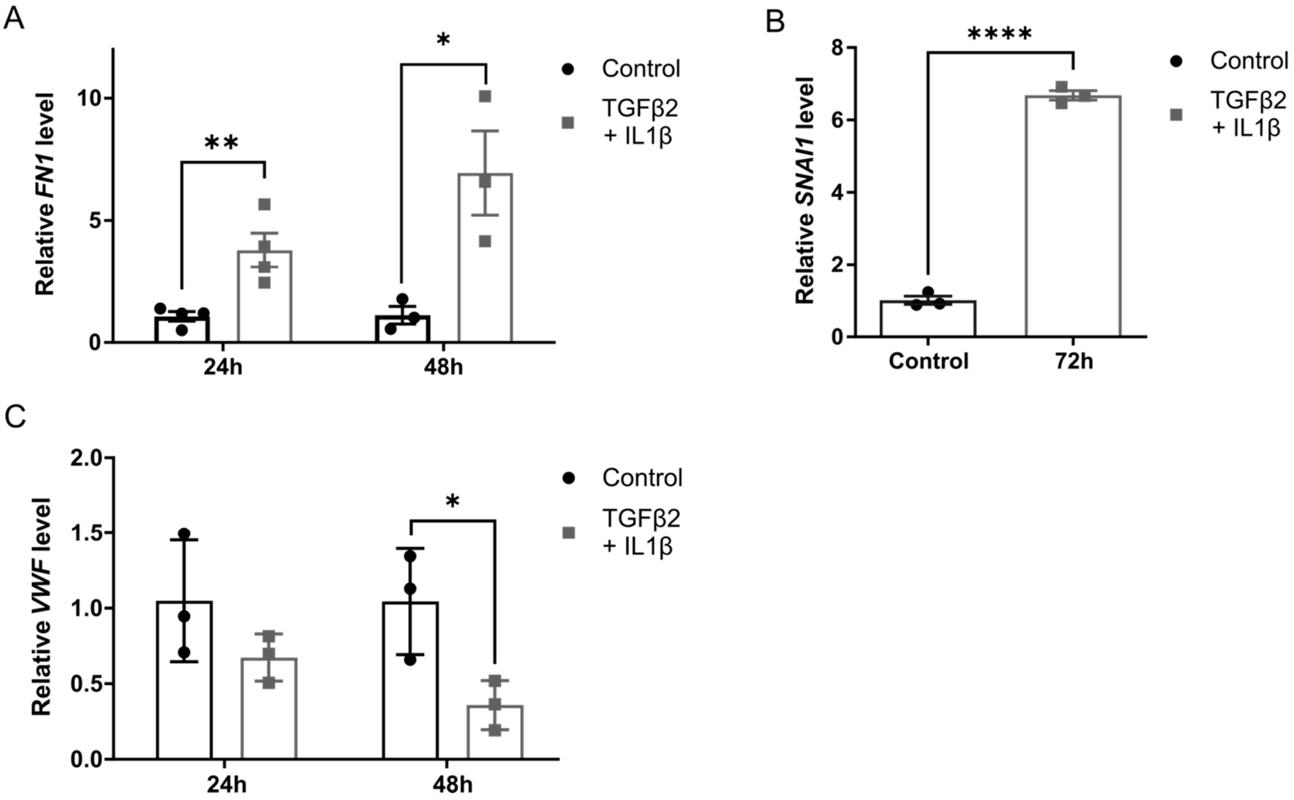

Figure S3. HMEC cells undergo endothelial to mesenchymal transition with TGFβ2 and IL1β treatment.

HMEC were treated with TGFβ2 (10 ng/mL) or TGFβ2 and IL1β (10 ng/mL). Gene expression level for fibronectin (A) and vWF (C) was assessed in endothelial cells at 24 and 48 h post-treatment with TGFβ2 and IL1β. (B) Snail expression was evaluated 72 h post-treatment with TGFβ2 and IL1β. Expression was normalised to the expression of the housekeeping gene Hprt1 and the error bars are representative of the standard error of the mean. (n = 3) Statistical significance was calculated by unpaired Student’s t-test and one way ANOVA. (* $p < 0.05$; ** $p < 0.01$; **** $p < 0.0001$).

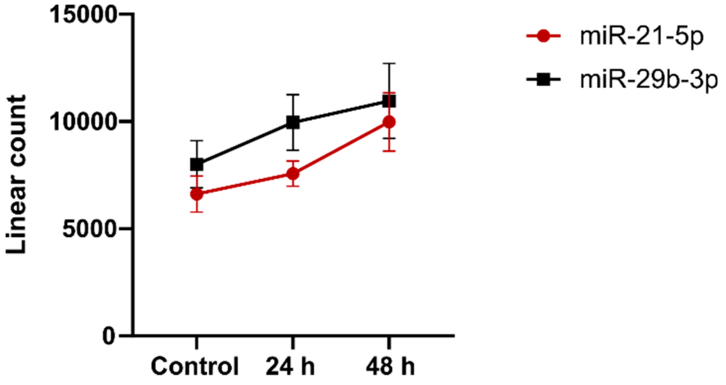

Figure S4. Linear count of miR-21-5p and miR-29b-3p in endothelial cells undergoing EndMT.

Endothelial cells were treated 24 and 48 h with TGFβ2 and IL1β to induce EndMT. MiR-21-5p and miR-29b-3p expression level was evaluated with a nCounter assay ($n = 2$).

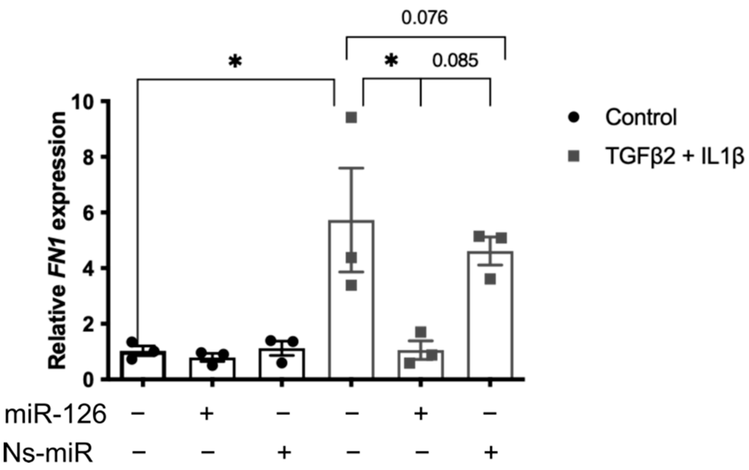

Figure S5. Gene expression of fibronectin after miR-126-3p transfection in HMECs undergoing EndMT.

HMECs were transfected with 50 nM of miR-126-3p mimics using lipofectamine followed by TGFβ2 and IL1β (10 ng/mL) treatment for 48 h. Transfection of non-specific miRNA (50 nM) was used as a control. The gene expression levels of fibronectin were assessed in endothelial cells post-transfection and post-treatment. Expression was normalised to the expression of the housekeeping gene Hprt1 and error bars are representative of the standard error of the mean. Statistical significance was assessed by one-way ANOVA followed by the Tukey test (* $p < 0.05$) ($n = 3$).

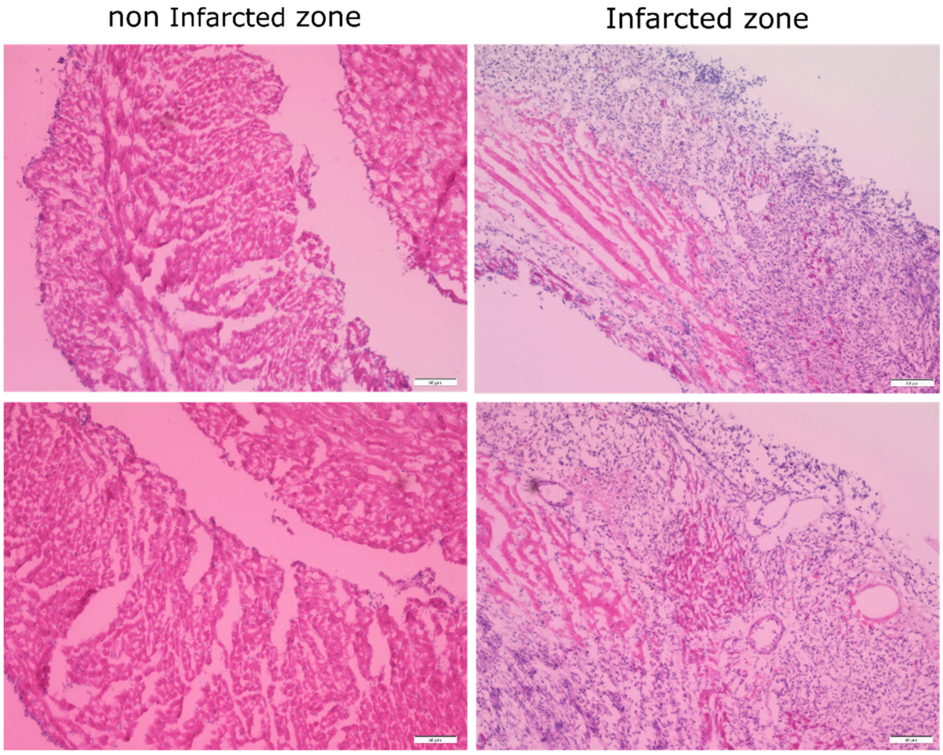

Figure S6. Haematoxylin-eosin staining of transverse sections of MI mice heart.

Infiltration of immune cells and the presence of fibrous tissue can be observed in the infarcted zone compared to the non-infarcted zone suggesting severe cardiac injury. Scale bars : 50µm.

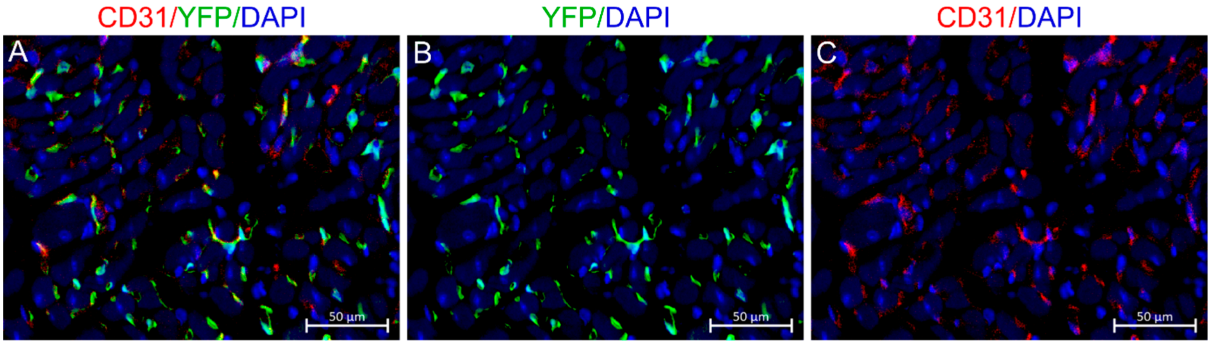

Figure S7. Lineage tracing of endothelial cells in the non-infarcted area of the mouse heart.

Staining for YFP (endothelial origin) (B) and CD31 (C) were performed on frozen sections of *Cdh5*-Cre;*Rosa26R*-YFP T/T mouse heart to evaluate the lineage tracing of endothelial cells (A). Tissues were counterstained with DAPI.

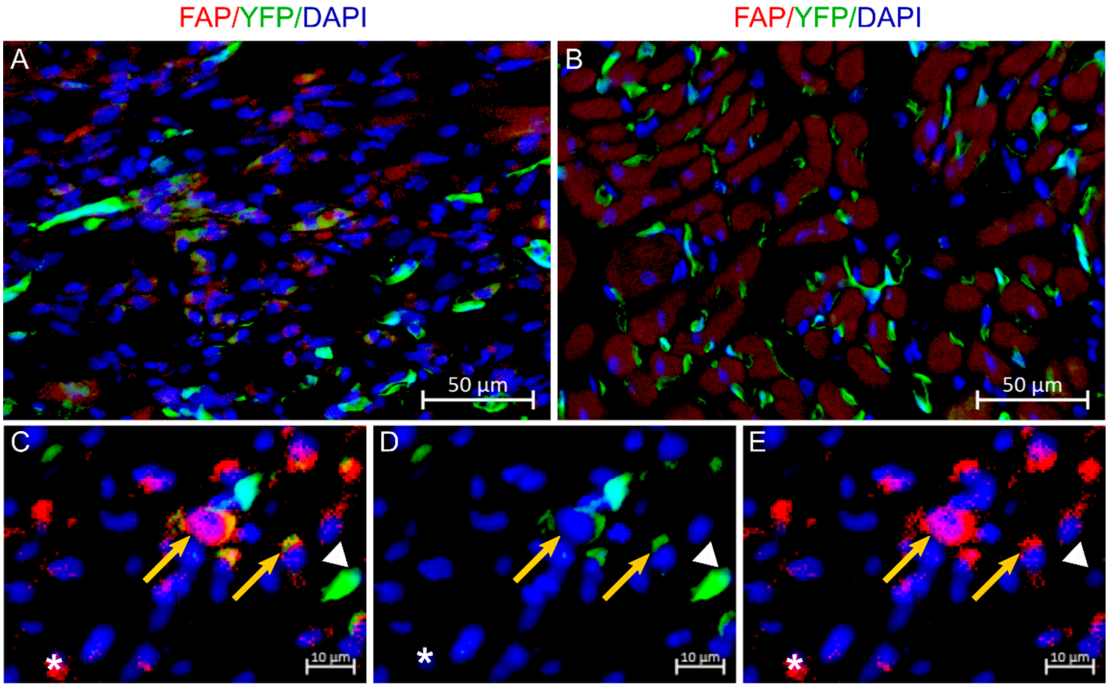

Figure S8. Endothelial-to-mesenchymal transition is present in fibrotic hearts.

Cdh5-Cre-ERT2;*Rosa26R*-stop-YFP mice underwent MI and were sacrificed 5 days later. Transverse sections of mouse heart post-MI were stained for FAP and YFP (endothelial cell origin). Micrographs show an infarcted area (A) and a non-infarcted area (B). A higher magnification of the infarcted area is shown in the lower panel (C,D,E) YFP+ FAP+ cells were indicated with a yellow arrow. Single YFP+ or FAP+ cells were designated by the white arrowhead or white star, respectively (n = 2).

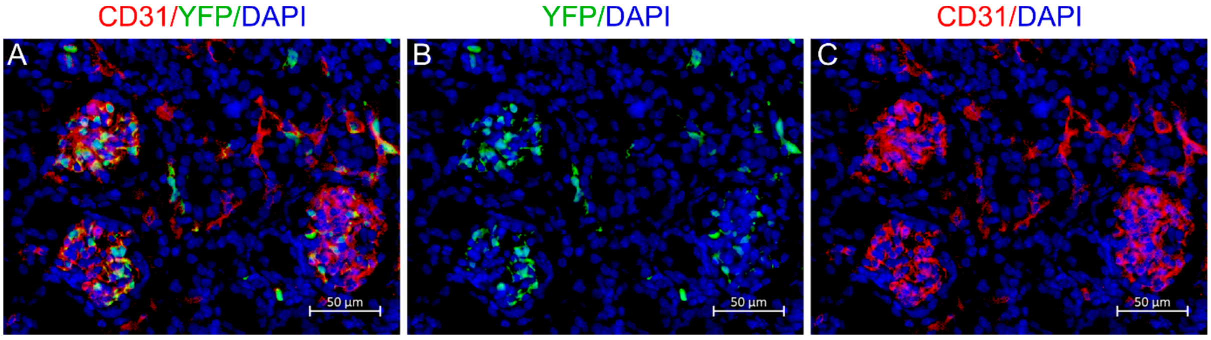

Figure S9. Lineage tracing of endothelial cells in healthy mouse kidney.

Cryosections of *Cdh5*-Cre; *Rosa26R*-YFP T/T mouse kidney were stained YFP (endothelial origin) (B) and for CD31 (C) to assess the accuracy of the endothelial lineage tracing (A). Nuclei were stained with DAPI.
